# Item-level reanalysis of DASH outcomes after flexor tendon repair using Svensson’s non-parametric method

**DOI:** 10.1186/s12891-026-09626-y

**Published:** 2026-02-27

**Authors:** Annika Dahlgren, Sara Chevalley, Joakim Strömberg, Anders Björkman

**Affiliations:** 1 Department of Occupational Therapy and Physiotherapy, Sahlgrenska University Hospital, Gothenburg, Sweden; 2 Department of Hand Surgery, Sahlgrenska University Hospital, Gothenburg, Sweden; 3 Institute of Clinical Sciences, Sahlgrenska Academy, University of Gothenburg, Gothenburg, Sweden

**Keywords:** Flexor tendon injuries, Evaluation study, Patient reported outcome measure, Rehabilitation

## Abstract

**Objectives:**

To examine longitudinal item-level changes in the Disabilities of the Arm, Shoulder and Hand (DASH) questionnaire after flexor tendon repair and to compare outcomes between passive mobilisation with place-and-hold exercises and true active mobilisation.

**Design:**

Randomised controlled trial with follow-ups at 3, 6, and 12 months postoperatively.

**Subjects/patients:**

Sixty-four patients were randomised before rehabilitation; 54 completed follow-up.

**Methods:**

DASH responses were reanalysed using Svensson’s non-parametric rank-based method to evaluate systematic group-level changes and individual variability at the item level.

**Results:**

Symptom items showed the greatest deterioration relative to baseline, with 37–69% of patients reporting persistent impairment at 12 months. Patients most often rated symptom items as ‘mild’ and ‘moderate’. All symptom items and three of four social functioning items demonstrated significant changes at each follow-up, whereas only 8 of 21 activity items were consistently significant. No statistically significant differences were observed between treatment groups.

**Conclusion:**

One year after flexor tendon repair, patients continued to report difficulties, particularly with symptoms (stiffness, weakness, pain) and social interaction, while activity limitations were less prominent. These challenges were consistent across the cohort and did not differ between mobilisation protocols. Item-level analysis provided insights beyond total DASH scores, highlighting the clinical value of this approach.

**Trial registration:**

The original randomized controlled trial was conducted in accordance with the CONSORT guidelines and was registered on ClinicalTrials.gov, PRS (Protocol Registration and Result System) (ClinicalTrials.gov Identifier NCT04385485 protocol ID 1001). The trial was retrospectively registered on 2017-07-04. The present study represents a secondary analysis, specifically an itemlevel reanalysis, of data from this trial.

**Supplementary Information:**

The online version contains supplementary material available at 10.1186/s12891-026-09626-y.

## Introduction

Flexor tendon injuries are common, with an annual incidence of 12 per 100,000 in men and 2 per 100,000 in women [1]. Standard treatment consists of primary repair with multi-strand core and epitendinous sutures, allowing for early mobilisation [2–4]. Postoperative rehabilitation is then crucial to prevent adhesions and joint stiffness and restore active finger motion [5–10]. Nevertheless, comparative studies of rehabilitation protocols after flexor tendon repair are limited and have yielded inconclusive results. Some studies support active mobilisation [9, 11], others recommend passive mobilisation with place-and-hold exercises [12], while several others report no significant differences between the two approaches [7, 13, 14]. Inconsistent terminology across protocols further complicates direct comparison [15]. A Cochrane review from 2021 concluded that evidence remains insufficient to determine the superiority of any current rehabilitation method following flexor tendon repair [16].

Objective functional outcomes after flexor tendon repair, such as range of motion or grip strength, do not always align with patients’ perceived activity levels, underscoring a potential gap between clinical assessments and patient experience [17]. To address this gap, patient-reported outcome measures (PROMs) are now routinely incorporated into evaluations following flexor tendon repair [18]. PROMs provide valuable insight into the patient perspective, often capturing aspects of recovery not fully reflected by clinician-based measures [19–21].

The Disabilities of the Arm, Shoulder and Hand (DASH) questionnaire is a widely used region-specific PROM for evaluating upper extremity function, including outcomes after flexor tendon repair [13, 22–24]. The questionnaire generates a 30-item total score ranging from 0 (no disability) to 100 (severe disability) [19]. However, different patterns of functional challenges may result in similar sum scores, limiting interpretability at the individual level [25]. While this total score enables comparisons across conditions and interventions [26], its relevance for specific diagnostic groups requires critical evaluation [27]. As the DASH consists of ordinal categorical items, summation may obscure important differences in patient experience. Svensson’s rank-invariant method addresses this limitation by separating systematic group change from individual variability [28–30], a distinction shown to be clinically meaningful when individual variation exceeds group trends [31]. This approach has been applied in various musculoskeletal conditions [32–36].

The primary aim of this randomised controlled trial was to examine longitudinal item-level changes on the DASH disability/symptom scale following flexor tendon repair. We used Svensson’s rank-invariant to explore whether individual DASH items adequately capture outcomes specific to flexor tendon repair. A secondary aim was to compare item-level outcomes between patients treated with passive mobilisation with place-and-hold exercises and those treated with true active mobilisation protocols.

## Material & methods

This prospective longitudinal study was conducted at the Department of Hand Surgery, Sahlgrenska University Hospital, Gothenburg, Sweden, as a follow-up to a randomised controlled trial by Chevalley et al. [13]. The original study design and outcomes at the 1- and 5-year follow-ups have been published [13, 37].

Briefly, between 2013 and 2017, patients with acute flexor tendon injury admitted to the department were screened for inclusion. Eligibility criteria included complete transection of the flexor digitorum profundus (FDP) tendon in digits 2–5 within zone 1 or 2, age > 16 years, and the ability to follow an early mobilisation protocol. Patients with concomitant flexor digitorum superficialis (FDS) and/or digital nerve injuries were eligible if other criteria were met. Exclusion criteria were severe concomitant injury in the same hand, bilateral flexor tendon repairs, distal zone 1 injuries requiring reinsertion, prior injury to the affected finger, inability to comply with early mobilisation, active substance abuse, or psychiatric disorder. All tendon repairs were performed using a standardised four-strand core suture with an epitendinous suture, providing sufficient strength to allow early mobilisation. Concomitant FDS or digital nerve injuries were repaired when present [13].

At the initial rehabilitation visit (1–3 days postoperatively), patients were randomised to either passive mobilisation with rubber bands and place-and-hold exercises or to true active mobilisation. Randomisation was performed using a computer-generated sequence, with allocation concealment ensured by sealed, consecutively numbered envelopes opened after surgery and before initiation of rehabilitation, as described by Chevalley et al. [13]. Due to the nature of the interventions, treating hand therapists and patients could not be blinded; however, outcome assessments at the 6- and 12-month follow-ups were performed by a blinded examiner. Rehabilitation was supervised by experienced hand therapists over 12 weeks, and progressive adjustments were made to the training program. Most restrictions were lifted at 12 weeks, except for heavy manual labour and gym activities, which were prohibited until 4 months postoperatively. Follow-up at the rehabilitation unit beyond 12 weeks was scheduled only if clinically indicated [13].

In this study, participants completed the DASH questionnaire [19] retrospectively at baseline during the initial rehabilitation visit, reflecting pre-injury status, and prospectively at 3, 6, and 12 months after surgery, enabling evaluation of patient-perceived recovery over 1 year. The 30-item disability/symptom scale of the DASH questionnaire evaluates the patient’s health status during the preceding week. Developed as a region-specific instrument by health professionals [38, 39], it includes 21 items assessing the difficulty experienced in performing physical activities, five items evaluating symptoms (pain, activity-related pain, tingling, weakness, stiffness), and four items addressing the impact of symptoms on social activities, work, sleep, and self-image. Each item is rated with five response categories.

### Statistics

As this was a secondary post-hoc exploration of a previously published trial of an existing cohort, the sample size was fixed and no a priori power calculation was performed. Analyses using Svensson’s method were descriptive and exploratory, and the results offer a nuanced perspective on patient-reported recovery trajectories. Descriptive analyses were performed using SPSS version 29.0.0.1. Changes in DASH disability/symptom items were evaluated with a previously described non-parametric, rank-based method for ordered categorical data [28–30, 40]. This approach enables assessment of both systematic group-level change and individual variability, offering a clinically meaningful evaluation of intervention effects. Analyses were conducted for the total cohort and for each treatment group separately. No imputation was applied for missing responses to preserve item-level integrity, leading to variable sample sizes across items. Missing data were due to dropouts from missed follow-up visits or incomplete questionnaires, including patients with tendon re-ruptures who did not complete DASH follow-up.

Systematic change was quantified using effect estimates derived from Svensson’s method, relative position (RP) and relative concentration (RC), with values ranging from − 1 to 1. An RP of 0 indicates no systematic shift, whereas positive or negative values reflect a shift toward higher or lower rated disability and symptoms, respectively. RC assesses redistribution of response categories, where a value of 0 denotes no concentration change and nonzero values indicate clustering toward specific ends of the scale. Individual variability not explained by systematic change was quantified using relative rank variance (RV), with values ranging from 0 (no variability) to 1 (maximum variability). The upper limit of RV depends on the number of response categories; for the DASH, which includes five categories, the theoretical maximum RV is 0.61. This parameter reflects the degree of heterogeneity in item-level changes for each individual patient [28, 29]. Lower RV values indicate more uniform response patterns, whereas higher values (typically > 0.20), as proposed by Svensson [41] suggest greater individual variability in the pattern of change.

Contingency tables were used to visualise both systematic and individual changes, with the main diagonal representing unchanged category responses. Marginal distributions were plotted as cumulative proportions to generate relative operating characteristic (ROC) curves. Curves above the diagonal indicate systematic improvement, curves below the diagonal indicate deterioration, and S-shaped curves reflect redistribution of responses, such as clustering toward milder or more severe disability or symptom categories.

All RP, RC, and RV values and their 95% confidence intervals (CIs) were calculated using freely available software [42]. Statistical significance was defined as a 95% CI excluding zero. Between-group differences were considered significant if their 95% CIs did not overlap [40]. Bonferroni correction was applied for multiple comparisons at an overall significance level of 5% [43].

## Results

A total of 64 patients were randomised to active mobilisation or passive mobilisation with place-and-hold exercises following flexor tendon repair. Ten patients (five in each group) were lost to follow-up, leaving 54 patients (26 active, 28 passive) in the final analysis. Reasons for dropout included three tendon ruptures in each group, one postoperative infection, and three non-attendance at follow-up visits. The median age of the cohort was 38 years (range 17–69), and 36 patients (67%) were men (Table 1).

**Table 1:** Demographics

Variable	Total study cohort *n* = 54	Active mobilisation *n* = 26	Passive mobilisation with place and hold *n* = 28
Age (y)			
Median (range)	38 (17-69)	41 (17-69)	37 (18-62)
Sex			
Female	18	10	8
Male	36	16	20
Occupation			
Employed	40	16	24
Student	8	6	2
Unemployed	1	1	0
Retired	5	3	2
Injury to dominant hand	25	9	16
Injured finger			
Dig 2	20	11	9
Dig 3	9	2	7
Dig 4	4	1	3
Dig 5	21	12	9
Concomitant injury			
FDS	22	12	10
Digital nerve	23	9	14
Zone			
1	15	7	8
2	39	19	20

At 3 months, stiffness (87%), weakness (79%), and difficulty opening a jar (69%) were the most frequently reported impairments. Pain-related symptoms were noted by 44–59% of patients. By 12 months, impairments persisted but at lower frequencies, with 69% still reporting stiffness, 55% weakness, and 37% difficulty opening a jar (Table 2). Impacts on symptoms and psychosocial items (items 22–30) persisted at 6 and 12 months, particularly stiffness, weakness, and limitations in daily activities. Stiffness was consistently the most prevalent symptom across all follow-ups (Table 3).

**Table 2:**
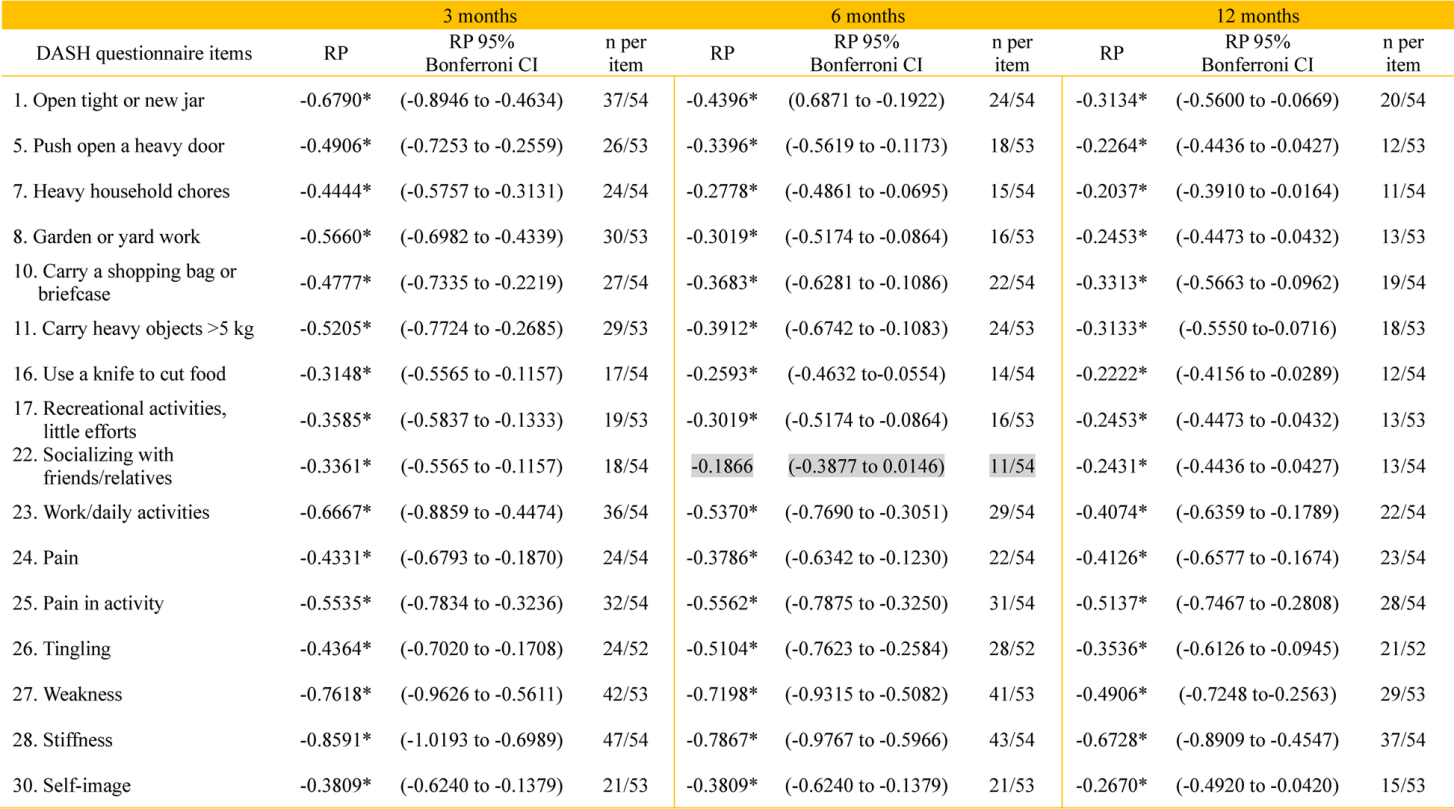
Items from the DASH disability/symptom scale showing statistically significant systematic group change (relative position, RP) in the total study cohort at 3, 6, and 12 months after flexor tendon repair. Results are presented with Bonferroni-corrected 95% confidence intervals (CI) and the number of patients per item

a. 95% confidence interval (CI) in brackets. b. Number of patients per item (n per item). c. * Statistically significant items. d. Non-statistically significant items marked in grey

**Table 3:** Proportion of patients with symptoms/total number of patients in (items 24-28 of the DASH questionnaire) at 3 months, 6 months and 12 months after flexor tendon repair, presented as number with symptoms/total number and percentage of patients

DASH questionnaire	3 months		6 months		12 months	
24. Arm, shoulder or hand pain	24/54	44%	22/54	41%	23/54	43%
25. Arm, shoulder or hand pain when you performed any specific activity	32/54	59%	31/54	57%	28/54	52%
26. Tingling (pins and needles) in your arm, shoulder or hand	24/52	46%	27/54	50%	21/54	39%
27. Weakness in your arm, shoulder or hand.	42/53	79%	41/53	77%	29/53	55%
28. Stiffness in your arm, shoulder or hand.	47/54	87%	43/54	80%	37/54	69%

**Fig. 1:**
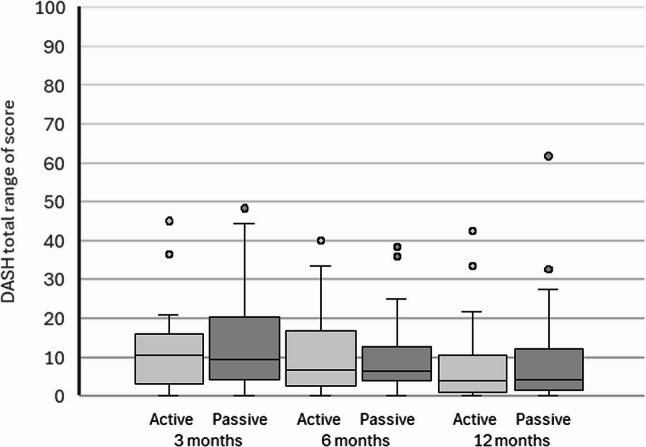
Distribution of the DASH sum score across time points after surgery and for the two treatment groups shown with the median, interquartile range, total range, and outliers

The results were similar between the treatment groups at all follow-ups, with no clear differences observed in the DASH item-level responses (Table 4). The analysis of DASH sum scores showed that median DASH scores were similar between groups at all time points. Variability, reflected by interquartile ranges, was higher in the passive group at 3 months than in the active group. However, this was reversed at 6 months. The levels were then comparable between groups at 12 months. Across follow-ups, there was no consistent pattern favouring either mobilisation protocol (Fig. 1 and Table 5). This temporal pattern may partly reflect postoperative restrictions, as patients were subject to activity limitations during the first 3 months after surgery. Consequently, functional use of the operated hand increased primarily between the 3- and 6-month follow-ups, in parallel with the expected biological healing process after flexor tendon injury [44], which may explain the observed improvement and reduced variability at 6 months.

**Table 4:**
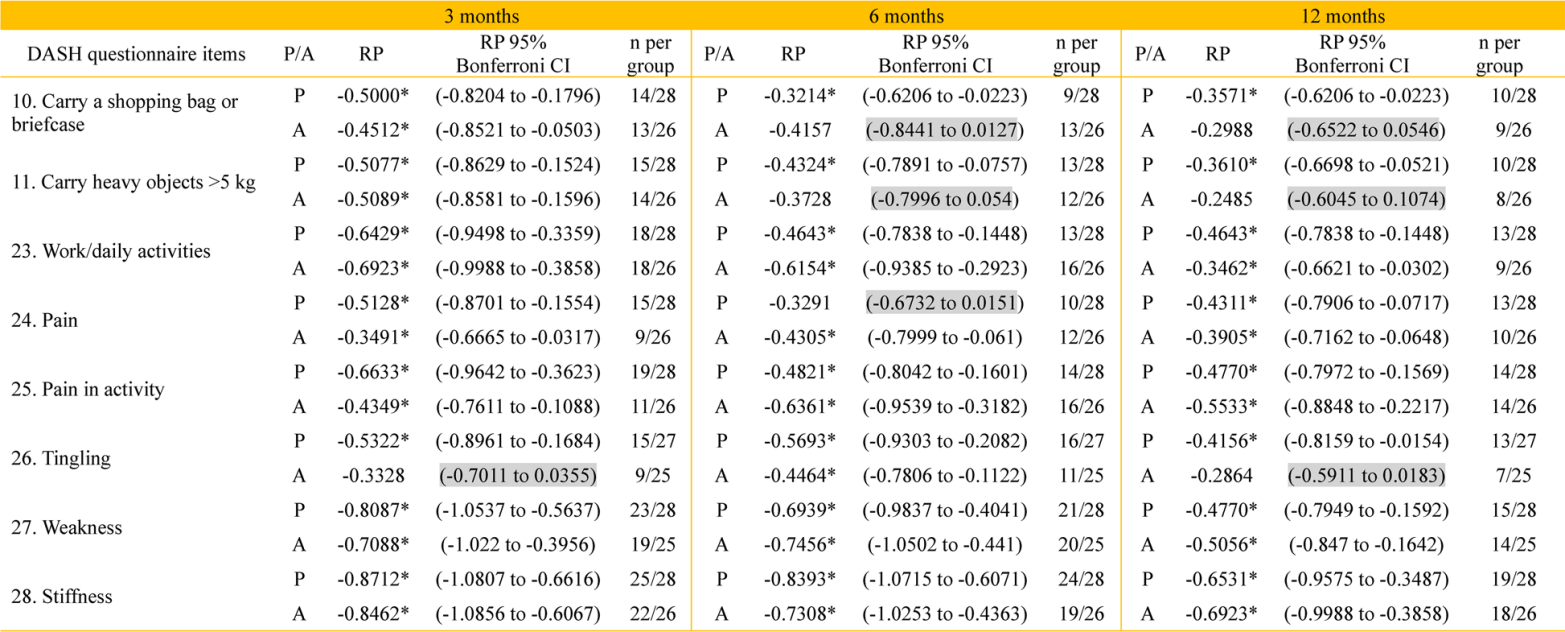
Items from the DASH disability/symptom scale showing statistically significant systematic group change (relative position, RP) in patients treated with passive mobilisation with place-and-hold (P) and active mobilisation (A) at 3, 6, and 12 months after flexor tendon repair. Results are presented with Bonferroni-corrected 95% confidence intervals (CI) and the number of patients per group

a. 95% confidence interval (CI) in brackets. b. Number of patients per mobilisation group (n per group). c. * Statistically significant items. d. Non-statistically significant items marked in grey

**Table 5:** DASH sum score analysis for the total cohort study and treatment group differences over time

	Total cohort study			Active mobilisation			Passive mobilisation		
	3 months	6 months	12 months	3 months	6 months	12 months	3 months	6 months	12 months
Md	11	7	4	11	8	5	11	7	4
IQR	13.7	11.5	10.3	12.7	14.1	10.5	16.9	8.3	10.8
Range	0-50	0-40	0-62	0-45	0-40	0-43	2-50	0-38	0-62

a. IQR, interquartile range, b. Md, Median, c. Values are given as (Md), (IQR), and DASH sum score range, where 0 = no disability and 100 = severe disability

**Fig. 2:**
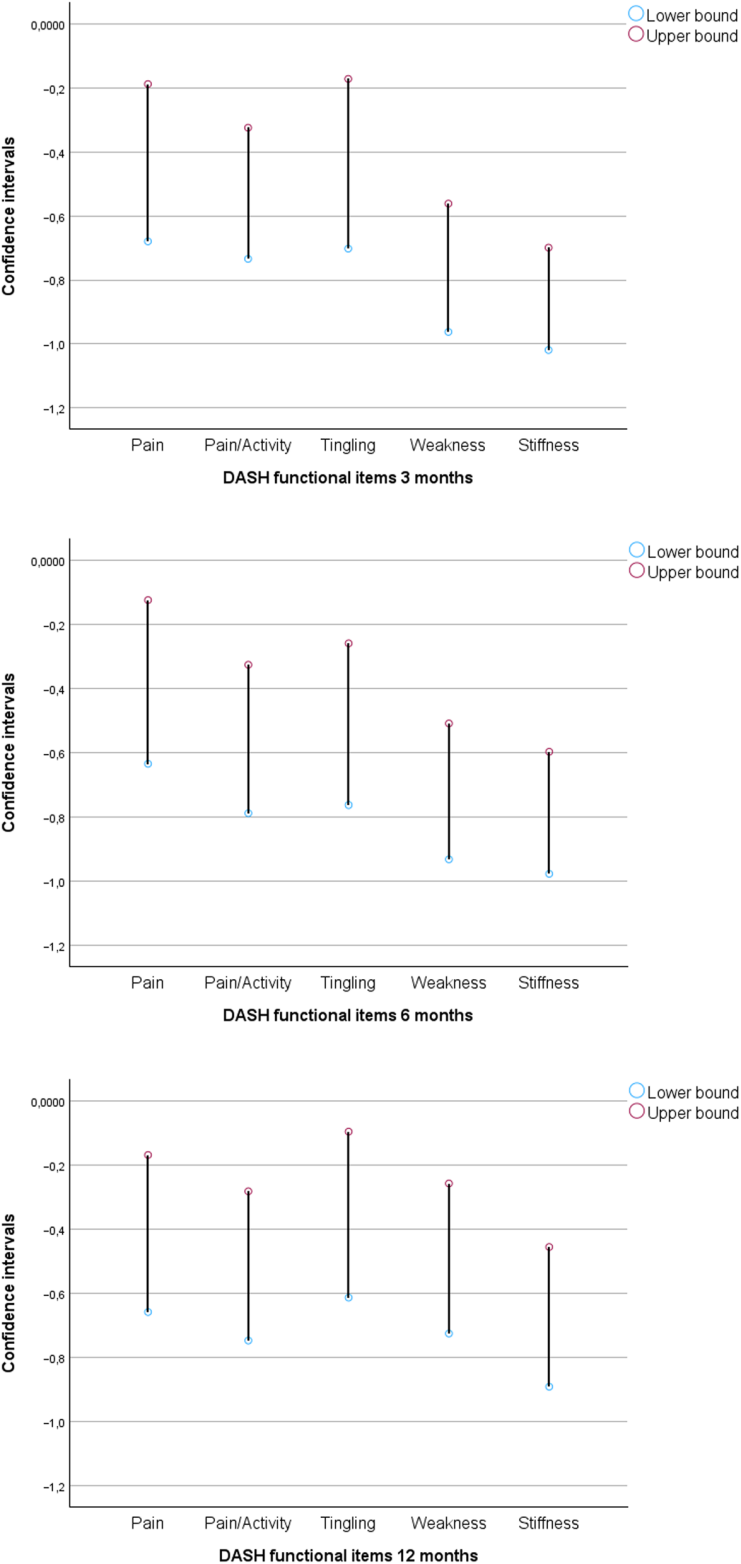
95% confidence intervals for Relative Position (RP) for the five DASH symptom items, derived using Svensson’s method. Negative values indicate deterioration in self-reported symptoms across follow-up

Item-level analysis showed that stiffness (item 28) and weakness (item 27) were the most persistent problems, with consistent negative shifts across all follow-ups (Table 3). Most patients rated stiffness and weakness as ‘mild’ or ‘moderate’. Stiffness showed the strongest and most sustained deterioration. Across all three follow-ups, stiffness consistently showed the largest negative relative position (RP) estimates, followed by weakness, indicating that these functional domains were most affected over time. At 3 months, RP ranged from − 0.86 (95% CI − 1.02 to − 0.70) for stiffness and − 0.76 (95% CI − 0.96 to − 0.56) for weakness, with comparatively narrow confidence intervals (Table 2). Although the magnitude of RP estimates decreased at 6 and 12 months, the relative ranking of items remained stable. Confidence intervals for stiffness and weakness were consistently narrower than for the other items, indicating greater precision of these estimates across follow-up (Fig. 2).

**Fig. 3:**
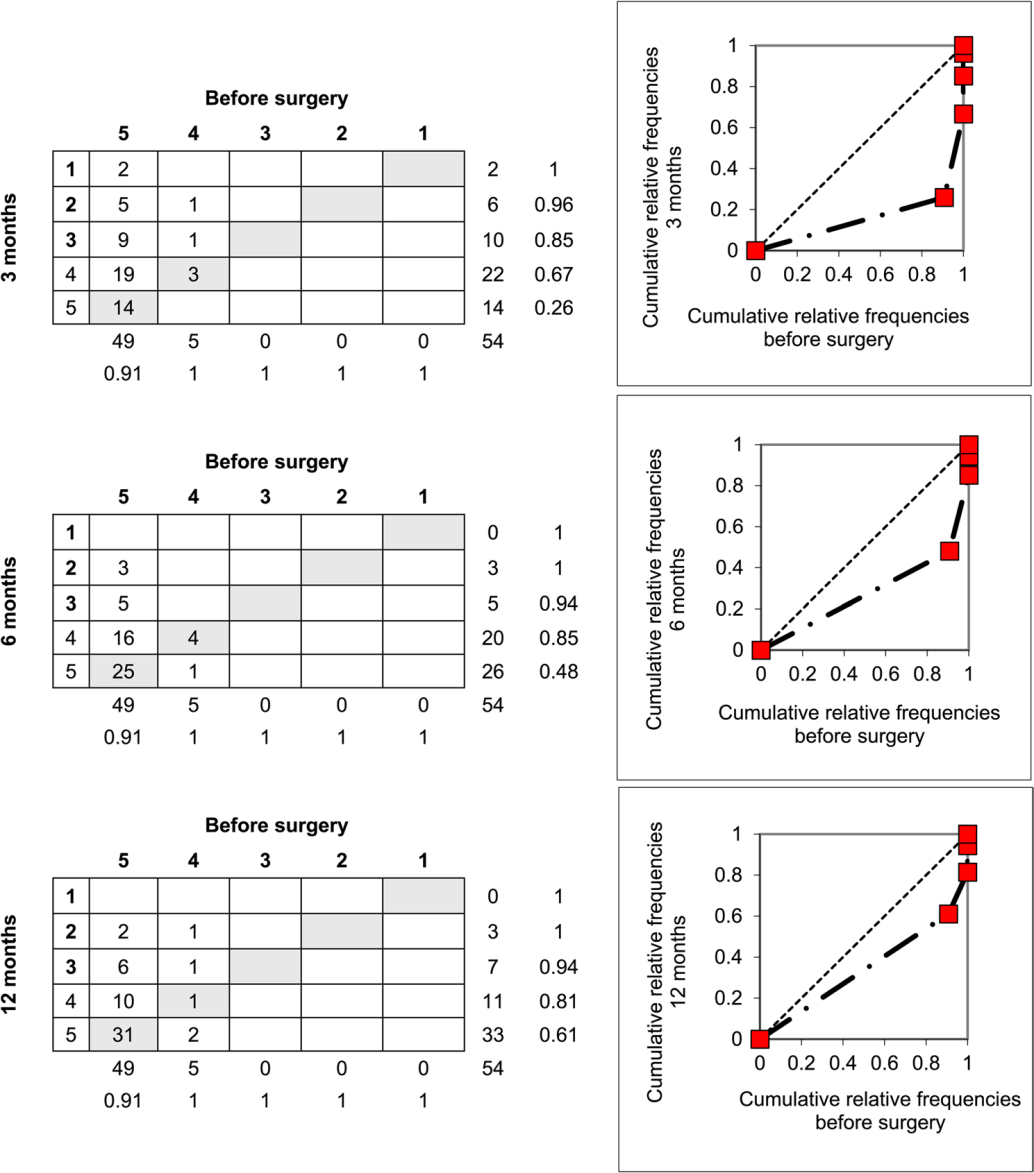
Contingency tables illustrating patient responses to item 1 (open a tight or new jar) of the DASH questionnaire, with response categories: 5 = unable, 4 = severe difficulty, 3 = moderate difficulty, 2 = mild difficulty, 1 = no difficulty, before surgery and at 3, 6, and 12 months after flexor tendon repair. Grey cells indicate unchanged responses. Marginal distributions show a systematic negative shift, with more patients reporting difficulty after surgery compared with baseline. The relative operating characteristic (ROC) curve illustrates this change: the diagonal line represents no difference from baseline, while curves below the diagonal indicate a systematic negative shift. The distance of the curve from the diagonal reflects the magnitude of this change, with clustering around the“mild” and “moderate” difficulty categories. Relative position (RP) values were–0.68, –0.44, and –0.31 across the 3-, 6-, and 12-month follow-ups, respectively (*n* = 54)

**Fig. 4:**
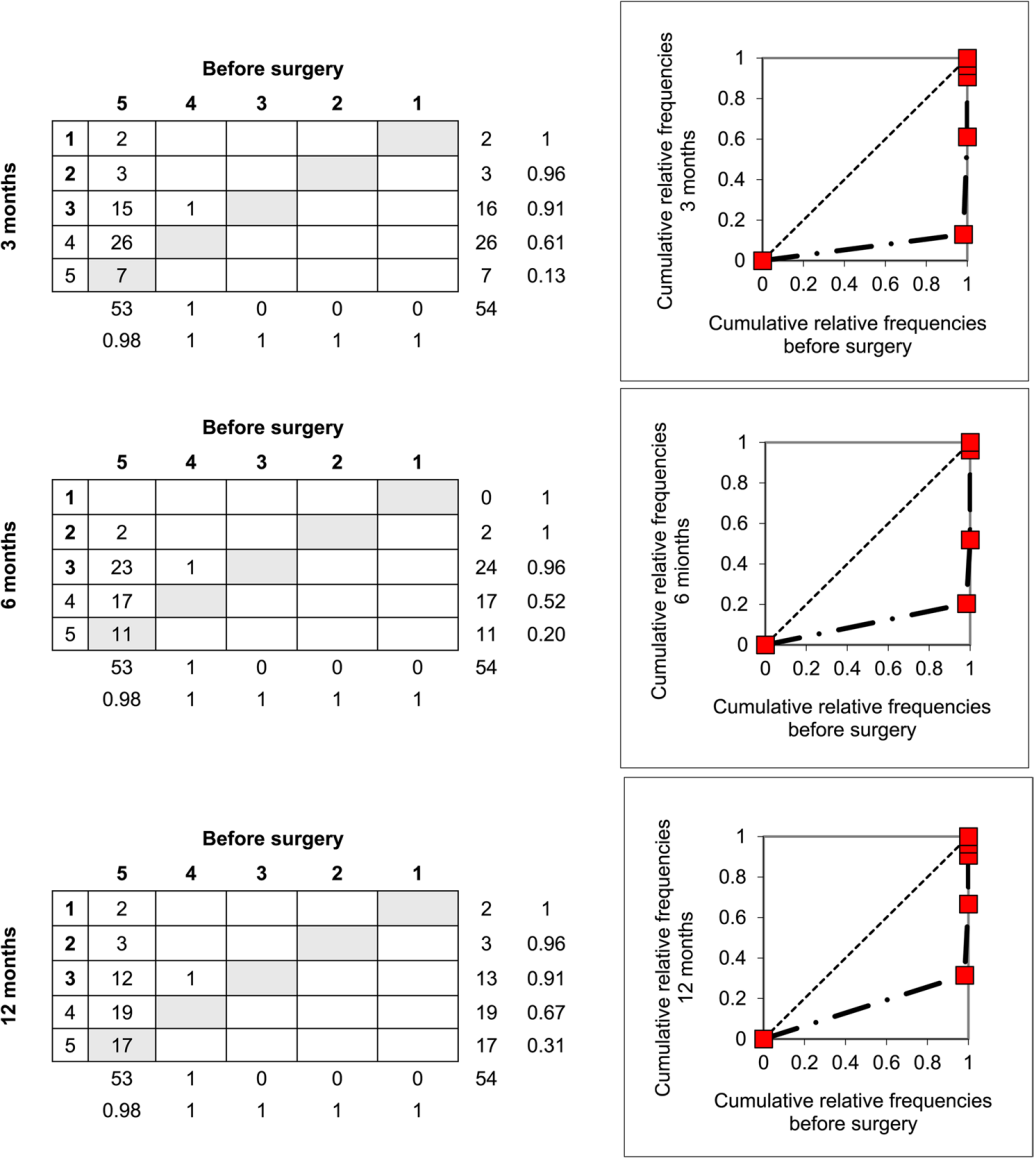
Contingency tables illustrating patient responses to item 28 (stiffness) of the DASH questionnaire, with response categories: 5 = extreme, 4 = severe, 3 = moderate, 2 = mild, 1 = none, before surgery and at 3, 6, and 12 months after flexor tendon repair. Grey cells indicate unchanged responses. Marginal distributions show a systematic negative shift, with more patients reporting stiffness after surgery compared with baseline. The relative operating characteristic (ROC) curve illustrates this deterioration: the diagonal line represents no change from baseline, while curves below the diagonal indicate a systematic negative shift. The distance of the curve from the diagonal reflects the magnitude of this change, with clustering around the “mild” and “moderate” categories. Relative position (RP) values were –0.86, –0.79, and –0.67 across the 3-, 6-, and 12-month follow-ups, respectively (*n* = 54)

Among the 21 activity-related items, only 8 demonstrated consistent change over time. Opening a jar (item 1) was notable for an initial clustering around ‘mild difficulty’ at 3 months, with gradual recovery by 12 months (61% reporting ‘no difficulty’) (Fig. 3). Of the four items related to social functioning, three (socializing, work/daily activities, and self-image) remained significantly affected at all follow-ups. Changes in all symptom-related items (pain, pain with activity, tingling, weakness, stiffness) were consistently significant at each time point (Table 3). For stiffness, patients were 67% more likely to report ongoing symptoms at 12 months compared with baseline, most often in the ‘mild’ or ‘moderate’ categories (Figs. 4).

Individual variability in responses was generally low across all DASH items, indicating that most patients showed similar patterns of change in activity, symptoms, and psychosocial outcomes. Given the five response categories in the DASH, the maximum possible variability is 0.61; the values observed in this study were well below this level (range 0.0 to 0.24), indicating limited variability in observed change patterns.

## Discussion

This study demonstrated that stiffness and weakness remained the most persistent problems reported by patients during the first year after flexor tendon repair, with stiffness (item 28) consistently showing the highest prevalence. Although median DASH scores declined over time, indicating functional improvement, item-level analysis revealed that a large proportion of patients continued to experience limitations in performing physically demanding activities, pain-related symptoms, and deficits in psychosocial domains. These findings highlight the value of examining PROMs at the item level, as total sum scores alone may obscure ongoing difficulties in daily life.

Our findings are consistent with earlier reports showing a disconnect between clinical measures such as range of motion and grip strength and the patient’s perception of recovery, particularly in relation to residual stiffness and weakness [13, 17, 23, 37]. Previous studies have emphasised that tendon healing often restores motion to a functional level, yet patients may continue to report symptoms that interfere with daily activities and quality of life [11, 18, 21].

In the analysis, we applied Svensson’s non-parametric method [28–30, 40], a rank-based method that respects the ordinal nature of the data and is more sensitive to detecting both systematic and individual changes. In contrast, most previous studies on flexor tendon repair have reported DASH sum scores, which assume equal spacing between ordinal response categories and may obscure important differences in response patterns. Thus, the current analysis provides a more nuanced picture of patient recovery by separating systematic group-level change from individual variability and enabling the identification of item-level improvements and deteriorations. The low relative rank variance observed across items suggests that most patients followed a similar recovery trajectory, indicating consistency in observed change patterns, while the identification of item-specific impairments such as stiffness, weakness, and pain highlights domains that remain under-recognised in routine reporting.

The use of confidence intervals added value by providing insight into both the magnitude and precision of the observed changes after surgery, consistent with an estimation-based approach to interpretation [45]. The estimated effects (RP) for the five DASH symptom items varied in both range and magnitude; however, all confidence intervals indicated a deterioration in symptom severity relative to baseline. Wider intervals for three of the five items reflect greater uncertainty, suggesting that these results should be interpreted with caution. In contrast, the items “weakness” and “stiffness” showed more negative and narrower confidence intervals, which increases confidence that these findings reflect a deterioration in symptoms and supports the interpretation that they may be clinically meaningful [46]. As Svensson’s method is based on relative ranking rather than quantitative score change and respects the ordinal nature of the data, clinically meaningful thresholds such as minimal clinically important difference (MCID) were not applied. Instead, an estimation-based interpretation using confidence intervals was adopted, providing clinically relevant information on patient-reported patterns of change.

Beyond the analytic method, the characteristics and performance of the PROM also warrant consideration. In this study, no total scores approached the upper limit of the DASH questionnaire. Response distributions suggested a concentration at the higher-functioning end of the DASH, indicating a possible ceiling effect, which may in part reflect that the questionnaire captures disability rather than handedness [47]. As the DASH assesses disability rather than hand-specific performance, patients may compensate by using the non-operated hand when performing daily activities, which may partly contribute to high functioning ratings and ceiling effects on activity items. This may suggest that the questionnaire has limited sensitivity in detecting more severe levels of disability in patients following flexor tendon repair. The five response categories may also restrict sensitivity, potentially masking subtle but clinically relevant differences between individuals or treatment groups [28, 29]. In addition, the absence of long-term between-group differences may partly reflect that the DASH captures overall disability rather than hand-specific performance, allowing compensatory strategies over time. Moreover, the DASH does not address certain critical domains after tendon repair, such as bilateral hand use and fine dexterity. Instruments like the Michigan Hand Outcomes Questionnaire (MHQ), which include domains for grip strength, dexterity, and bimanual tasks [48, 49], may offer greater specificity in future research. As this study was based on a reanalysis of existing data, the DASH was the only available patient-reported outcome measure, precluding the inclusion of hand-specific PROMs or objective functional assessments of hand function. This constraint limits the interpretability of functional outcomes, particularly with respect to fine motor impairment, and should be considered when interpreting the results.

The QuickDASH, an abbreviated 11-item version of the DASH, was developed to reduce respondent burden and improve the feasibility of data collection in clinical and research settings [50]. Both instruments are widely used after flexor tendon repair, although the QuickDASH is often favoured in registry-based and large cohort studies because of its efficiency [13, 50]. However, key items showing substantial postoperative change in our cohort—particularly stiffness and weakness—are absent from the QuickDASH. This raises concerns about the sensitivity of the questionnaire for this patient group, especially as flexor tendon repairs were not included in the original validation cohorts. In contrast, the DASH captures these domains and permits item-level analysis, as demonstrated in our study. Altogether, these findings highlight the need to balance feasibility with content validity when selecting PROMs for patients recovering from flexor tendon repair.

Regarding the two rehabilitation protocols, differences in the item-level analysis were most evident at the 3-month follow-up. At this time point, patients in the passive mobilisation group reported more negative changes in activity and symptom items than those in the active mobilisation group. This is consistent with prior evidence suggesting that early mobilisation may accelerate initial recovery [9, 11, 12]. However, these differences diminished by 6 months and were negligible at 12 months, aligning with other trials reporting no long-term advantage of one mobilisation strategy over the other [7, 13, 15]. Together, these findings highlight the importance of considering the timing of assessment—early functional gains with active protocols may influence the short-term perception of recovery, while long-term outcomes may be similar regardless of the mobilisation approach.

This study has several strengths, including its prospective design, multiple assessment time points with repeated use of PROMs, and application of a statistical method suited to ordinal data, which enabled detailed analysis of item-level recovery patterns. The main limitations are the modest sample size, which restricted subgroup analyses. Given the post-hoc nature of the analysis and the fixed sample size, the absence of between-group differences should be interpreted with caution, as the study was not powered to detect small or moderate group effects. Although retrospective baseline assessments collected close to the injury have been shown to be reasonably reliable [51], such assessments may still affect the precision of estimated change over time. The baseline was therefore primarily used to contextualize recovery trajectories rather than as a strict inferential baseline.

In addition, outcomes were not linked to objective measures such as range of motion or grip strength, which may limit generalisability. Nevertheless, the consistent item-level patterns observed support the internal validity of the findings. Another limitation concerns the analytic method. The non-parametric rank-based approach provides greater sensitivity to item-level changes and has been successfully applied in other musculoskeletal conditions [28–30, 40]. However, its use reduces comparability with studies employing DASH sum score analysis, including interpretations based on the numerical minimal clinically important difference MCID. While offering new insights into patient-reported outcomes after flexor tendon repair, its relative unfamiliarity may challenge broader acceptance. The present analyses were therefore intended to complement, rather than replace, established DASH sum score analyses. Validation in larger and more diverse patient cohorts is needed to support its integration into standard outcome evaluation frameworks.

In summary, this study shows that stiffness, weakness, and pain remain the most significant patient-perceived problems after flexor tendon repair, even as overall DASH scores improve. These findings highlight the importance of selecting outcome measures that are both psychometrically robust and sensitive to the specific challenges of this patient population. Item-level analysis can reveal persisting problems that may otherwise be masked by composite scores, supporting the continued use of region-specific PROMs in rehabilitation research. Incorporating appropriate PROM-based monitoring into clinical care may help guide individualised rehabilitation strategies and align treatment goals more closely with patient priorities.

## Supplementary Information

Supplementary Material 1: CONSORT 2010 checklist of information to include when reporting a randomised trial.

Supplementary Material 2: Consort Flow chart Chevalley 2022.

## Data Availability

The de-identified dataset analysed during the current study is available from the corresponding author on reasonable request. Data will be shared in accordance with the ethical approval and applicable data protection regulations, and may require additional ethical approval where appropriate.

## Abbreviations

DASH: Disabilities of the Arm, Shoulder and Hand questionnaire
PROM: Patient-Reported Outcome Measure
FDP: Flexor Digitorum Profundus
FDS: Flexor Digitorum Superficialis
SPSS: Statistical Package for the Social Sciences
RP: Relative Position
RC: Relative Concentration
RV: Relative rank Variance
ROC: Relative Operating Characteristic curve
CI: Confidence interval
MCID: Minimal Clinically Important Difference
MHQ: Michigan Hand Outcomes Questionnaire
QuickDASH: Quick Disabilities of the Arm, Shoulder and Hand questionnaire
