# Supplementary figures and images for: Item-level reanalysis of DASH outcomes after flexor tendon repair using Svensson’s non-parametric method
Source: BMC Musculoskelet Disord. 2026 Feb 27;27:292. doi: 10.1186/s12891-026-09626-y (PMC13064354; doi:10.1186/s12891-026-09626-y)

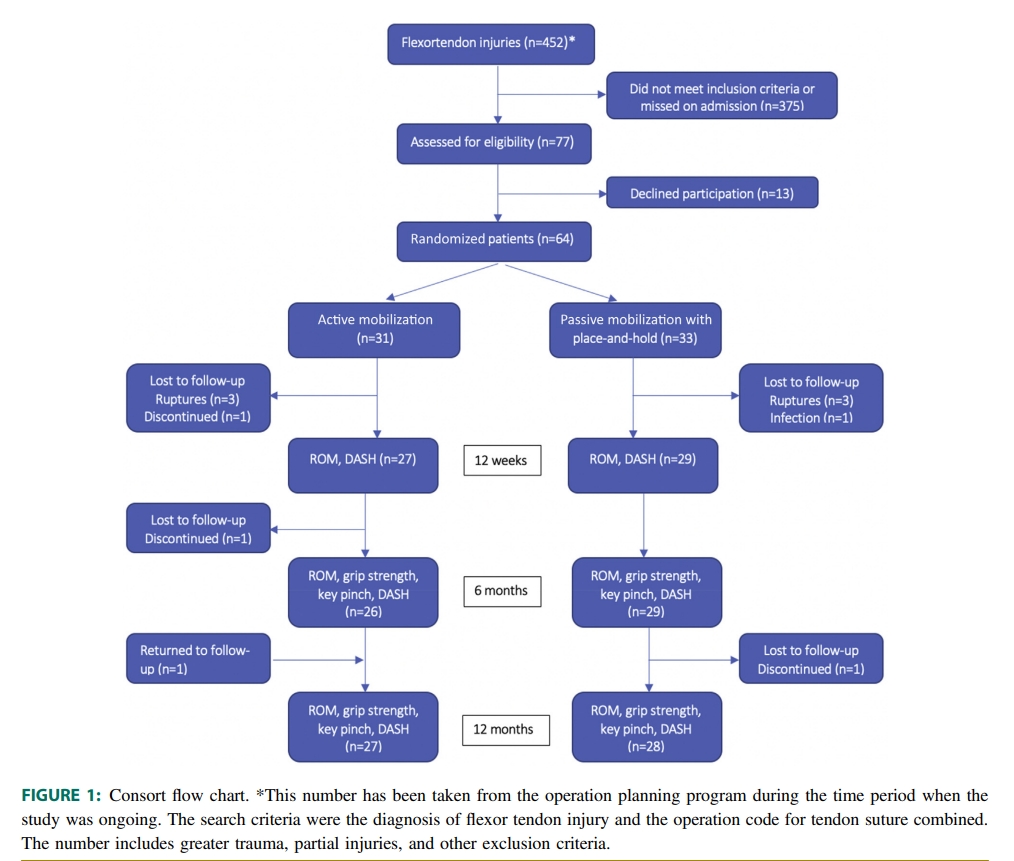

Supplement: Supplementary file 2 — Supplementary Material 2: Consort Flow chart Chevalley 2022. [file 12891_2026_9626_MOESM2_ESM.jpeg]
